# Serotonin and Dopamine Receptor Expression in Solid Tumours Including Rare Cancers

**DOI:** 10.1007/s12253-019-00734-w

**Published:** 2019-09-02

**Authors:** Marloes A. M. Peters, Coby Meijer, Rudolf S. N. Fehrmann, Annemiek M. E. Walenkamp, Ido P. Kema, Elisabeth G. E. de Vries, Harry Hollema, Sjoukje F. Oosting

**Affiliations:** 1 Department of Medical Oncology, University Medical Center Groningen, University of Groningen, Hanzeplein 1, Box 30.001, 9700 RB Groningen, The Netherlands; 2 Department of Laboratory Medicine, University Medical Center Groningen, University of Groningen, 9700 RB Groningen, The Netherlands; 3 Department of Pathology, University Medical Center Groningen, University of Groningen, 9700 RB Groningen, The Netherlands

**Keywords:** Serotonin receptor 1B, Serotonin receptor 2B, Dopamine receptor D2, Dopamine receptor D1, Neovascularization, Neoplasms

## Abstract

In preclinical studies serotonin stimulates and dopamine inhibits tumour growth and angiogenesis. Information regarding serotonin and dopamine receptor (5-HTR and DRD) expression in human cancers is limited. Therefore, we screened a large tumour set for receptor mRNA overexpression using functional genomic mRNA (FGmRNA) profiling, and we analysed protein expression and location of 5-HTR1B, 5-HTR2B, DRD1, and DRD2 with immunohistochemistry in different tumour types. With FGmRNA profiling 11,756 samples representing 43 tumour types were compared to 3,520 normal tissue samples to analyse receptor overexpression. 5-HTR2B overexpression was present in many tumour types, most frequently in uveal melanomas (56%). Receptor overexpression in rare cancers included 5-HTR1B in nasopharyngeal carcinoma (17%), DRD1 in ependymoma (30%) and synovial sarcoma (21%), and DRD2 in astrocytoma (13%). Immunohistochemistry demonstrated high 5-HTR2B protein expression on melanoma and gastro-intestinal stromal tumour cells and endothelial cells of colon, ovarian, breast, renal and pancreatic tumours. 5-HTR1B expression was predominantly low. High DRD2 protein expression on tumour cells was observed in 48% of pheochromocytomas, and DRD1 expression ranged from 14% in melanoma to 57% in renal cell carcinoma. In conclusion, 5-HTR1B, 5-HTR2B, DRD1, and DRD2 show mRNA overexpression in a broad spectrum of common and rare cancers. 5-HTR2B protein is frequently highly expressed in human cancers, especially on endothelial cells. These findings support further investigation of especially 5HTR2B as a potential treatment target.

**Electronic supplementary material:**

The online version of this article (10.1007/s12253-019-00734-w) contains supplementary material, which is available to authorized users.

## Introduction

Serotonin and dopamine are biogenic amines, which are produced in the central nervous system and gastrointestinal tract. Throughout the body, they are transported by platelets. Serotonin and dopamine play a role in vascular tone, gastrointestinal motility, limb movement control and other physiological processes [1, 2]. Preclinical studies discovered that serotonin and dopamine also influence tumour angiogenesis and tumour growth [3–10].

Angiogenesis is one of the hallmarks of cancer. It is a prerequisite for tumour growth as it secures oxygen and nutrient supply and removal of break-down products from the tumour microenvironment [11]. Serotonin stimulates tumour angiogenesis via activation of serotonin receptor 1B (5-HTR1B) and serotonin receptor 2B (5-HTR2B) [3–5]. Serotonin can also directly stimulate tumour cell proliferation via various serotonin receptors on tumour cells [10], whereas depletion of serotonin in mice with murine melanoma and lung tumours resulted in slower growth compared with mice having normal serotonin concentrations [5]. Research demonstrated that dopamine inhibits angiogenesis and thereby tumour growth in animal models of colon cancer, ovarian cancer, and breast cancer via activation of dopamine receptor D2 (DRD2) [6, 7]. On the opposite, it was shown that the DRD2 pathway is activated in human pancreatic cancer and that growth of pancreatic cancer xenografts was inhibited by DRD2 antagonists in mice [12]. Contradictory results have also been published for dopamine receptor D1 (DRD1), as both inhibition as well as stimulation of tumour growth has been reported upon receptor activation in animal models of ovarian cancer and breast cancer [6, 8, 9].

Serotonin and dopamine receptor agonists and antagonists are prescribed for the treatment of Parkinson’s disease, psychosis, nausea, and migraine [1, 13, 14], but have not been explored for an anti-tumour effect in cancer patients. Information regarding serotonin and dopamine receptor expression in human cancers is limited. Therefore, we screened for 5-HTR1B, 5-HTR2B, DRD1, and DRD2 mRNA overexpression in a large dataset with common and rare tumour types using functional genomic mRNA (FGmRNA) profiling and we determined protein expression and localization of the serotonin and dopamine receptors 5-HTR1B, 5-HTR2B, DRD1, and DRD2 in eight tumour types.

## Materials and Methods

### FGmRNA Profiling

RNA microarray expression data of 11,756 human tumour samples were collected from Gene Expression Omnibus (GEO), a large publicly available data set [15]. From these expression data, FGmRNA profiles were created. Detailed information about FGmRNA profiling was described previously [16]. In short, principal component analysis is used to identify major regulators of the mRNA transcriptome. These so-called transcriptional components are used to correct the mRNA expression data for non-genetic differences, such as physiological and metabolic factors. The expression signal that remains after correction represents variance in mRNA expression due to genetic alterations. This is called the FGmRNA profile.

To determine a threshold for overexpression, FGmRNA profiling of 3,520 normal human tissue samples was performed, and the 97.5th percentile for 5-HTR1B, 5-HTR2B, DRD1 and DRD2 was calculated (see Supplementary Table 1 for an overview of non-cancer samples). For all tumour types, the percentage of samples with overexpression was calculated for each of the four receptors. Only tumour types with >10 samples available were used for this calculation.

### Immunohistochemistry

#### Patient Material

Selection of tumour types for immunohistochemistry was based on results from preclinical studies (colon cancer, ovarian cancer, breast cancer, pancreatic cancer, melanoma) [5–8, 12] or tumour characteristics (high vascularity for renal cell carcinoma, dopamine production for pheochromocytoma, and serotonin-induced proliferation of precursor cells for gastrointestinal stromal tumours (GIST)) [17, 18].

Formalin-fixed paraffin-embedded primary tumour tissues of colon cancer (*n* = 12), ovarian cancer (*n* = 12), breast cancer (*n* = 12), renal cell carcinoma (*n* = 14), and pancreatic cancer (*n* = 12) were used. A minimum of 12 tumours were selected per tumour type, because if 0/12 tumour samples show expression, the chance that it is relevant in >10% of patients is low. Furthermore, 3 tissue microarrays (TMA) were used, namely a melanoma TMA containing 36 tumour samples, a pheochromocytoma TMA containing 63 tumour samples, and a GIST TMA containing 76 tumour samples. The TMAs contained 3 cores with a diameter of 0.6 mm per patient.

Tissue samples used in this study were archival material. According to the Dutch Medical Research Involving Human Subjects act, no approval of the Institutional Review Board was required.

#### Immunohistochemical Procedure and Analysis

Formalin-fixed, 4 μm thick paraffin-embedded sections and TMAs were deparaffinized and rehydrated. Antigen retrieval was performed using heated citrate buffer (pH 6.0) for 5-HTR1B, 5-HTR2B, DRD1, and DRD2 or tris/EDTA buffer (pH 9.0) for CD31 for 15 min. For DRD2, slides were blocked with phosphate-buffered saline (PBS; pH 7,4) plus 0.1% Tween-20 for 20 min at room temperature. Endogen peroxidase was blocked in all slides with 1% H_2_O_2_ in PBS for 30 min at room temperature. For 5-HTR1B, additional avidin/biotin (avidin/biotin blocking kit (SP-2001); Vector Laboratories, Brunschwig Chemie, Amsterdam, The Netherlands) and human serum blocks were performed. The sections were incubated with the primary antibody for 1 h at room temperature for 5-HTR2B, DRD1, and CD31 or overnight at 4 °C for 5-HTR1B and DRD2. Primary antibodies used were mouse monoclonal 5-HTR1B 1:100 (clone 499,325; MAB5858, R&D systems, Abingdon, United Kingdom), mouse monoclonal 5-HTR2B 1:1000 (clone H-11; sc-376,834, Santa Cruz Biotechnology, Bio-Connect, Huissen, The Netherlands), mouse monoclonal DRD2 1:100 (clone B-10, sc-5303, Santa Cruz Biotechnology), rat monoclonal DRD1 1:75 (clone 1–1-F11 s.E6; D2944, Sigma Aldrich, Zwijndrecht, The Netherlands), and mouse monoclonal CD31 1:50 (clone JC70A; IR610, DAKO, Glostrup, Denmark). Subsequently, tissue sections were incubated with secondary antibodies (1:100 dilution or in case of 5-HTR1B a 1:300 dilution; all from DAKO). Between steps, slides were washed with PBS or in case of DRD2 with PBS plus 0.1% Tween-20. Staining was visualized using 3,3′-diaminobenzidine (DAB) and hematoxylin for counterstaining. Positive and negative controls (including immunoglobulin class-matched control sera) were included for each staining. Colon, testis, and prostate tissue served to validate the stainings as positive and negative controls. In addition standard hematoxylin & eosin (H&E) stainings were performed to evaluate tissue morphology**.**

#### Analysis of Immunohistochemistry

After immunohistochemical staining, slides were digitally scanned using the NanoZoomer (Hamamatsu Photonics, Shizuoka, Japan) and were scored using accompanied NanoZoomer Digital Pathology (NDP) software. All slides were scored by two independent investigators (M.P. and C.M. or H.H.) and compared to assure a minimal inter-observer difference. As an extra control, random slides were also evaluated by a pathologist (H.H.).

Tissue morphology was evaluated using H&E-stained slides. Staining intensity of the tumour cells and percentage of positive tumour cells was scored. Staining intensity of tumour cells was scored as negative (0), low (1), moderate (2), or high (3). The percentage of positive tumour cells was scored as no positive cells (0), 1–4% positive cells (1), 5–24% positive cells (2), 25–49% positive cells (3), 50–74% positive cells (4), and 75–100% positive cells (5). Receptor expression was scored using an immunoreactive score (IRS) to account for the heterogeneous staining in the section slides [19]. The IRS was defined by multiplying the staining intensity (category 0–3) with the percentage of positive tumour cells (category 0–5), creating a range from 0 to 15. Receptor expression was considered negative if IRS was 0, low if the IRS was 1 to 5, and high if the IRS was 6 to 15. For TMAs, at least two evaluable cores had to be present per tumour in order to consider it a representative score.

Receptor expression on tumour-associated blood vessels was only assessed on the whole tissue sections, as blood vessels were only in limited numbers or not at all present in the TMA samples due to their small diameter. Serotonin and dopamine receptor expression was scored by staining intensity (ranging from 0 to 3). Receptor expression was considered negative if intensity was 0, low if intensity was 1, and high if intensity was 2 or 3, CD31 staining was performed to confirm localization of tumour vessels.

## Results

### Overexpression of Serotonin and Dopamine Receptors Analysed with FGmRNA Profiling

For the frequency of overexpression of serotonin and dopamine receptors per tumour type, see Table 1.

**Table 1:** Overexpression of serotonin and dopamine receptors in different tumour types determined with functional genomic mRNA analysis

	Percentage overexpression (%)			
Tumor type (N)	5-HTR1B	5-HTR2B	DRD1	DRD2
Breast cancer				
ER-/Her2+ (455)	6.6	3.5	2.0	1.1
ER+/Her2- (1,678)	3.4	4.5	1.0	0.6
ER+/Her2+ (506)	3.8	4.5	1.4	0.6
TNBC (737)	7.3	3.5	0.7	1.2
CNS malignancies				
Anaplastic astrocytoma (36)	5.6	8.3	2.8	0.0
Anaplastic oligodendroglioma (26)	3.8	0.0	0.0	0.0
Astrocytoma (24)	4.2	25.0	4.2	12.5
Ependymoma (156)	1.3	0.0	29.5	0.6
Glioblastoma (389)	1.8	2.1	1.3	0.3
Medulloblastoma (148)	2.7	4.1	0.7	6.8
Meningioma (122)	3.8	7.6	0.0	2.5
Oligodendroglioma (23)	4.3	13.0	4.3	4.3
Pilocytic astrocytoma (135)	0.7	3.0	0.0	0.0
Endocrine malignancies				
Adrenal neuroblastoma (96)	4.2	5.2	1.0	25.0
Adrenocortical carcinoma (20)	0.0	10.0	0.0	0.0
Anaplastic thyroid carcinoma (21)	0.0	14.3	0.0	0.0
Papillary thyroid carcinoma (51)	2.0	7.8	0.0	0.0
Gastrointestinal malignancies				
Colorectal cancer (2,710)	1.3	4.3	0.9	3.7
Esophageal adenocarcinoma (41)	4.9	14.6	7.3	0.0
Esophageal squamous cell carcinoma (56)	0.0	3.6	0.0	0.0
Gastric cancer (332)	3.9	12.0	7.8	0.6
Hepatocellular carcinoma (364)	3.3	19.5	6.0	0.0
Pancreatic cancer (81)	0.0	8.6	1.2	0.0
Genitourinary malignancies				
Bladder cancer (39)	5.1	7.7	7.7	2.6
Chromophobe renal cancer (37)	0.0	0.0	0.0	2.7
Clear cell renal cancer (225)	5.8	7.6	0.9	1.8
Papillary renal cancer (37)	0.0	0.0	0.0	0.0
Prostate cancer (308)	3.6	5.2	0.6	1.9
Gynaecological malignancies				
Cervical cancer (62)	1.6	6.5	0.0	3.2
Ovarian cancer (187)	0.5	3.7	0.5	0.0
Head and neck cancer				
HNSCC (344)	4.9	0.6	2.0	1.2
Nasopharyngeal carcinoma (42)	16.7	11.9	0.0	2.4
Lung cancer				
Adenocarcinoma (1,019)	1.6	7.8	2.7	0.5
Squamous cell carcinoma (405)	0.2	2.7	1.5	0.5
Small cell lung cancer (103)	1.0	15.5	4.9	8.7
Melanoma				
Cutaneous (398)	3.8	9.0	0.0	1.3
Uveal (106)	2.8	55.7	2.8	0.9
Sarcoma				
Ewing’s sarcoma (26)	3.8	0.0	0.0	3.8
Leiomyosarcoma (60)	1.7	6.7	5.0	5.0
Liposarcoma (76)	3.9	19.7	1.3	1.3
Osteosarcoma (26)	0.0	15.4	0.0	0.0
Primitive neuroectodermal tumor (22)	4.5	0.0	0.0	4.5
Synovial sarcoma (34)	0.0	0.0	20.6	0.0
Undifferentiated sarcoma (95)	1.1	9.5	1.1	0.0

N; number, 5-HTR1B; serotonin receptor 1B, 5-HTR2B; serotonin receptor 2B, DRD1; dopamine receptor D1, DRD2; dopamine receptor D2, ER; estrogen receptor, Her2; human epidermal growth factor receptor 2, TNBC: triple negative breast cancer, CNS; central nervous system, HNSCC; head and neck squamous cell carcinoma

5-HTR1B was overexpressed in a low percentage of tumour samples per tumour type. The highest percentage was found in nasopharyngeal carcinoma: in 7 of 42 (17%) samples.

Of the four receptors, 5-HTR2B was most frequently overexpressed, especially in uveal melanomas (59 of 106 (56%) samples). Also in certain brain tumours and sarcomas, and in hepatocellular carcinoma and non-small cell lung cancer, a relatively high proportion of tumour samples showed overexpression of 5-HTR2B: 6 out of 24 astrocytomas (25%), 15 out of 76 liposarcomas (20%), 4 out of 26 osteosarcomas (15%), 71 out of 346 hepatocellular carcinomas (20%) and 16 out of 103 small cell lung cancers (16%).

Overexpression of DRD1 was most frequently found in ependymoma and synovial sarcoma with 46 out of 156 (30%) respectively 7 out of 34 (21%) of the samples.

DRD2 was most frequently overexpressed in adrenal neuroblastoma (24 out of 96 (25%) samples) and astrocytoma (3 out of 24 (13%) samples).

### Serotonin and Dopamine Receptor Expression Analysed with Immunohistochemistry

**Fig. 1:**
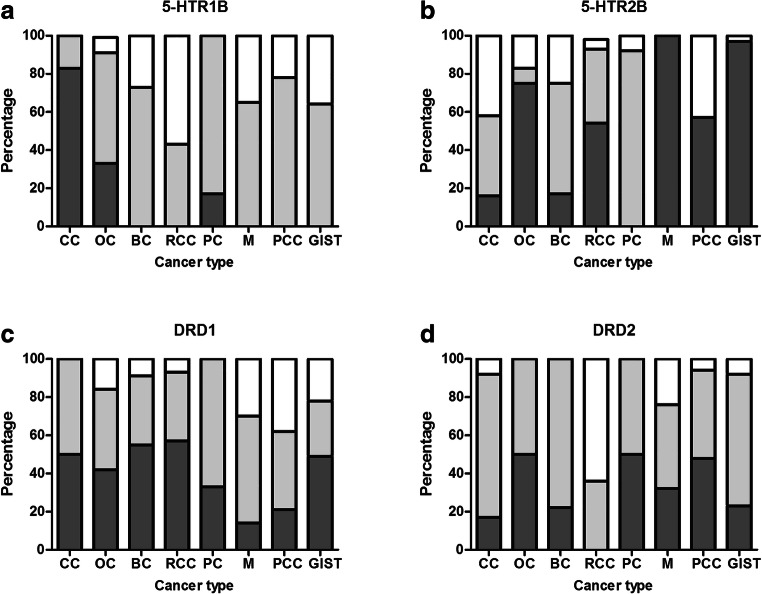
**a** Serotonin receptor 1B (5-HTR1B), **b** serotonin receptor 2B (5-HTR2B), **c** dopamine receptor D1 (DRD1), **d** and dopamine receptor D2 (DRD2) expression on tumour cells of colon cancer (CC) (*n* = 12), ovarian cancer (OC) (*n* = 12), breast cancer (BC) (*n* = 12), renal cell carcinoma (RCC) (*n* = 14), pancreatic cancer (PC) (*n* = 12), melanoma (M) (*n* = 36), pheochromocytoma (PCC) (*n* = 63) and gastrointestinal stromal tumours (GIST) (*n* = 76) as analysed with immunohistochemistry. Immunoreactive score (IRS) was used to classify receptor expression on tumour cells in three categories: negative (in white), low (in light grey) or high (in dark grey)

Tumour cells predominantly had low or absent 5-HTR1B expression, except for 10 out of 12 colon cancers and 4 out of 12 ovarian cancers showing high 5-HTR1B expression. 5-HTR2B was highly expressed on tumour cells of all melanomas and all but 2 GIST. Pheochromocytomas showed either absent or very high expression of 5-HTR2B on tumour cells. In some pheochromocytomas (*n* = 12), also small foci of strongly positive tumour cells were observed within a field of negative tumour cells. DRD1 was highly expressed on tumour cells of approximately 50% of colon cancers, ovarian cancers, breast cancers, renal cell carcinomas and GIST samples. For melanoma and pheochromocytoma, this was the case in less than 25% of samples. High DRD2 expression by tumour cells was most frequently observed in pheochromocytomas (30 out of 63 samples), ovarian cancer (6 out of 12 samples) and pancreatic cancer (6 out of 12 samples) (Fig. 1).

**Fig. 2:**
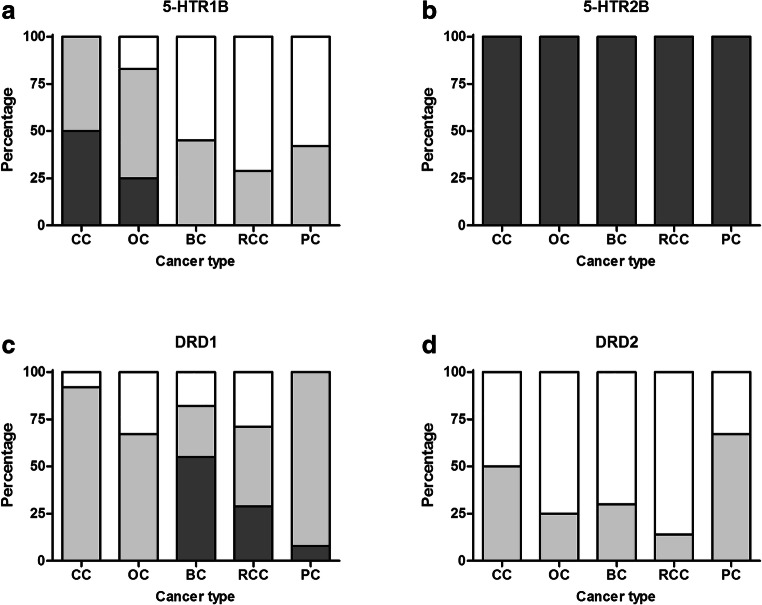
**a** Serotonin receptor 1B (5-HTR1B), **b** serotonin receptor 2B (5-HTR2B), **c** dopamine receptor D1 (DRD1), **d** and dopamine receptor D2 (DRD2) expression on endothelial cells of colon cancer (CC) (*n* = 12), ovarian cancer (OC) (*n* = 12), breast cancer (BC) (*n* = 12), renal cell carcinoma (RCC) (*n* = 14), and pancreatic cancer (PC) (*n* = 12) as analysed with immunohistochemistry. Receptor expression was classified by staining intensity. Receptor expression was considered negative if intensity was 0 (white), low if intensity was 1 (in light grey), and high if intensity was 2 or 3 (in dark grey)

5-HTR1B expression was predominantly low or absent on endothelial cells, except for 6 out of 12 colon cancers and 4 out of 12 ovarian cancers. 5-HTR2B, on the other hand, was highly expressed on endothelial cells of all tumour types investigated (colon, ovarian, breast, renal, and pancreatic cancer). Expression of DRD1 on endothelial cells varied per tumour type. In breast cancer, DRD1 was highly expressed on endothelial cells of 7 out of 12 samples. For the other tumour types, high expression of DRD1 on endothelial cells was only observed in 4 out of 14 renal cell carcinomas and 1 pancreatic cancer sample. DRD2 expression was low or absent on endothelial cells (Fig. 2).

**Fig. 3:**
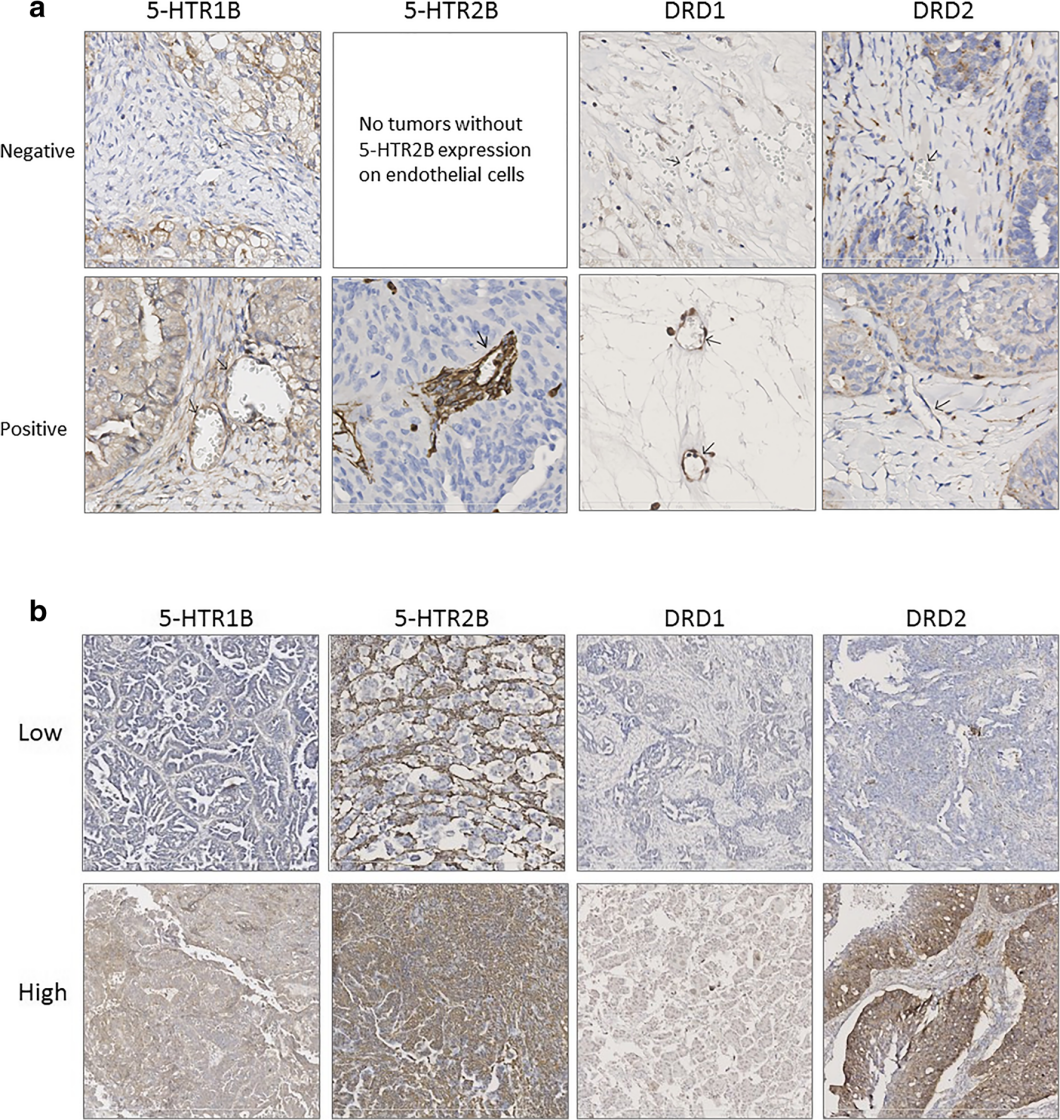
**a** Representative images of low and high expression of serotonin receptor 1B (5-HTR1B), serotonin receptor 2B (5-HTR2B), dopamine receptor D1 (DRD1), and dopamine receptor D2 (DRD2) by tumour cells in ovarian cancer (10x magnification). **b** Representative images of negative and positive (low or high) expression of serotonin receptor 1B (5-HTR1B), serotonin receptor 2B (5-HTR2B), dopamine receptor D1 (DRD1), and dopamine receptor D2 (DRD2) on blood vessels in ovarian cancer (40x magnification). Arrowheads indicate blood vessels that express the receptor of interest

For representative images of serotonin and dopamine receptor staining on ovarian cancer cells and tumour blood vessels, see Fig. 3a and b.

## Discussion

To our knowledge, this is the first study evaluating 5-HTR1B, 5-HTR2B, DRD1, and DRD2 (over)expression in a broad range of tumour types with both FGmRNA profiling and immunohistochemistry. We found that 5-HTR2B is more frequently overexpressed compared to 5-HTR1B, DRD1, and DRD2. All four receptors, however, are expressed across tumour types including rare cancers. 5-HTR2B is highly expressed on tumour cells in all melanomas and on tumour endothelial cells of colon, ovarian, breast, renal, and pancreatic cancer samples.

One of the great advantages of FGmRNA is that it enabled us to screen overexpression of serotonin and dopamine receptors in a large number of samples, leading to interesting findings in rare cancers like brain tumours and sarcomas. FGmRNA profiling identified mRNA overexpression based on a strictly chosen cut-off level, being receptor expression higher than the 97.5th percentile of expression in normal tissue (16). This may have led to the relatively low overexpression percentages found with FGmRNA profiling compared with the number of samples with high expression found with immunohistochemistry.

FGmRNA profiling and immunohistochemistry both demonstrated high 5-HTR2B expression in (uveal) melanoma. Two in vitro studies evaluated the effect of serotonin on melanoma growth with opposite results. Serotonin inhibited proliferation of 5-HTR2B expressing human melanoma cell line IPC298 but it did not affect B16F0 murine melanoma cell proliferation, in which however, receptor expression was not assessed [5, 20]. On the other hand, mice with serotonin depletion due to knockout of a serotonin transporter had smaller B16F0 murine melanomas than mice with a functional serotonin transporter and thus unaffected serotonin concentrations in blood [5].

5-HTR2B was highly expressed on endothelial cells of five tumour types evaluated using immunohistochemistry (colon cancer, ovarian cancer, breast cancer, renal cell carcinoma, and pancreatic cancer). This was in concordance with studies from a research group from Malmö, Sweden, which demonstrated 5-HTR2B protein expression by endothelial cells in 29 ovarian cancer and 102 breast cancer samples [21, 22]. In preclinical studies, the effect of 5-HTR2B antagonists on angiogenesis was evaluated: phosphorylation of serotonin-induced endothelial nitric oxide synthase (eNOS) was blocked in human umbilical vein endothelial cells (HUVEC) and in a murine lung cancer model, and was associated with decreased tumour microvessel density [5].

Preclinical research demonstrated that dopamine inhibits tumour angiogenesis via activation of DRD2. To our knowledge, this is the first study evaluating DRD2 expression on vasculature of human tumours. Previous studies demonstrated DRD2 protein expression on vessels of mouse ears and HUVEC [6]. In our study however, we observed low or absent DRD2 protein expression on tumour-associated endothelial cells. On tumour cells of 30/63 pheochromocytomas, DRD2 protein was highly expressed. This was in concordance with smaller studies in 10 respectively 39 pheochromocytomas [23, 24]. Interestingly, a phase I study with the DRD2 antagonist ONC201, demonstrated some clinical benefit in five endometrial and prostate cancer patients from a group of 27 advanced cancer patients [25]. However, a phase II study with ONC201 in 17 recurrent glioblastoma patients was closed after interim analysis since the target of a six month progression-free survival in 30% of patients was not reached [26]. In both studies, DRD2 expression was not evaluated. If the patient population could have been enriched based on target expression remains therefore unclear. Pituitary adenoma is a tumour type known to express DRD2, and the DRD2 agonist bromocriptine is already part of the standard treatment regimen in prolactin-producing adenomas [1, 27]. Dependent on tumour type and DRD2 expression on tumour cells, treatment with a DRD2 agonist or antagonist may have anti-tumour activity. For anti-angiogenic treatment however, the significance of DRD2 agonists seems limited based on our results regarding expression of this receptor on tumour-associated blood vessels.

In conclusion, serotonin and dopamine receptors are differentially (over)expressed in various tumour types by tumour and endothelial cells. Of these, 5-HTR2B is expressed most frequently. This study demonstrates that selection of patients with tumours of different backgrounds but with similar receptor expression profiles is possible. This could offer interesting future possibilities for basket studies. Basket studies include different tumour types and select patients with the same tumour characteristic for targeted treatment [28]. This allows to study new treatment modalities in rare tumours, such as brain tumours and sarcomas, for which there are currently limited treatment options.

## Electronic supplementary material

ESM 1 (DOCX 14 kb)
