# Supplementary material for: Serotonin and Dopamine Receptor Expression in Solid Tumours Including Rare Cancers
Source: Pathol Oncol Res. 2019 Sep 2;26(3):1539–47. doi: 10.1007/s12253-019-00734-w (PMC7297821; doi:10.1007/s12253-019-00734-w)
Supplement: Supplementary file 1 — (DOCX 14 kb) [file 12253_2019_734_MOESM1_ESM.docx]

| **Supplementary Table 1.** Number of samples per healthy tissue type used for functional genomic mRNA profiling | |
| --- | --- |
| **Tissue type** | **Number of samples** |
| B lymphocytes | 139 |
| Brain | 882 |
| Breast | 135 |
| Colorectal | 432 |
| Esophagus | 32 |
| Heart | 33 |
| Liver | 87 |
| Lung | 314 |
| Muscle | 484 |
| Nasal cavity | 74 |
| Nasopharynx | 7 |
| Oral cavity | 172 |
| Oropharynx | 14 |
| Pancreas | 19 |
| Parotid gland | 13 |
| Pharynx | 4 |
| Prostate | 53 |
| Sinuses | 9 |
| Skin | 495 |
| Spleen | 23 |
| Stomach | 42 |
| Thyroid gland | 57 |
